# Effect of antibiotics on Kupffer cell immunometabolism relative to intracellular killing of *S. aureus* using NAD(P)H fluorescence lifetime imaging

**DOI:** 10.1128/mbio.02124-25

**Published:** 2025-09-30

**Authors:** Brent Beadell, Annie Wong-Beringer

**Affiliations:** 1 Alfred E. Mann School of Pharmacy and Pharmaceutical Sciences, University of Southern California 5116, Los Angeles, California, USA; 2 Department of Pharmacy, Huntington Hospital 24815, Pasadena, California, USA; University of Pretoria, Pretoria, Gauteng, South Africa

**Keywords:** *Staphylococcus aureus*, antibiotics, vancomycin, ceftobiprole, daptomycin, tedizolid, Kupffer cells, bloodstream infections, immunometabolism, macrophages

## Abstract

*Staphylococcus aureus* bacteremia (SAB) remains a significant clinical burden with high mortality rates. Kupffer cells (KCs) are integral to clearing *Staphylococcus aureus* (SA) from the bloodstream yet also serve as a pathogenic reservoir, driving persistence and treatment failure. KCs utilize their metabolic plasticity to coordinate successful antimicrobial responses, and this process may be therapeutically targeted. We investigated the immunometabolic effects of antistaphylococcal antibiotics (vancomycin, ceftobiprole, daptomycin, and tedizolid) on KCs relative to clearance of intracellular SA through gene expression analysis and optical metabolic imaging using fluorescence lifetime imaging microscopy. We observed differential effects of antibiotics on KC polarization and metabolic signatures, with ceftobiprole inducing the strongest pro-inflammatory M1 expression profile and shortest mean NAD(P)H fluorescence lifetime in KCs in the absence of infection, and a retained M1 expression profile but longest NAD(P)H lifetime during infection. Daptomycin and tedizolid most effectively cleared intracellular SA, induced significantly longer NAD(P)H lifetimes, and exhibited decreased M1 gene expression. Vancomycin failed to control intracellular infection showing blunted M1 expression without altering NAD(P)H lifetimes. Stratification of NAD(P)H lifetime signals revealed daptomycin significantly increased binding to PDH-like enzymes and nitric oxide synthase 2 compared to other antibiotics. These findings reveal novel insights into antibiotic modulation of KC immunometabolism during SA infection, which may influence treatment outcomes in SAB.

**IMPORTANCE:**

*Staphylococcus aureus* bloodstream infection is a leading cause of sepsis and is associated with up to 30% mortality. Despite treatment with guideline-recommended antibiotics, persistent bacteremia develops in one in three patients, which may be attributed in part to survival of bacteria inside liver-resident macrophages known as KCs. Using gene expression and optical metabolic profiling, we demonstrated that antistaphylococcal antibiotics differentially affect KC metabolism and function, thereby contributing to the overall killing of intracellular bacteria. Daptomycin, ceftobiprole, and tedizolid exert distinct effects on KC metabolism that correspond to effective intracellular killing and prompt resolution of inflammatory response. Vancomycin, however, did not affect KC metabolism and was unable to control bacterial growth inside the cells. These findings suggest that choosing antibiotics based on direct antimicrobial activity as well as indirect effects on host immune function could improve treatment outcomes for patients with *S. aureus* bloodstream infection.

## INTRODUCTION

*Staphylococcus aureus* (SA) bloodstream infections (*Staphylococcus aureus* bacteremia [SAB]) remain one of the most common and lethal hospital-acquired infections in the United States, with mortality rates up to 40% despite receipt of antibiotic therapy (1–4). Persistent bacteremia develops in nearly a third of patients with SAB, with a 16% increase in mortality risk for each additional day of positive blood culture as shown in a large study involving over 800 patients by our group (5). This clinical challenge underscores the need to better understand host-pathogen interactions to optimize therapeutic approaches.

The liver serves as a sentinel immune organ in bloodstream infections, acting as a critical interface between the pathogen and early host immune response. This host defense function is provided by Kupffer cells (KCs), which are liver-resident macrophages that play a pivotal role in the initial capture and clearance of circulating pathogens (6, 7). However, Surewaard et al. recently described the role of KCs in providing a pathogenic reservoir in SAB, potentially contributing to persistence and treatment failure (8). Murine models of SAB have shown that within minutes of introduction into the bloodstream, KCs phagocytose roughly 90% of circulating SA, but a small proportion can survive and persist intracellularly within KCs to drive persistent infection and dissemination of SA to distal sites within the body (7–9). This dual role of KCs in both defending and harboring SA highlights the complexity of KC-pathogen interactions in determining infection outcomes in SAB.

Macrophages exhibit exceptional functional plasticity in response to stimuli originating from the local microenvironment such as pathogen-associated molecular patterns (PAMPs) and damage-associated molecular patterns. Through a process defined as polarization, macrophages become activated into a spectrum of different morphological and functional profiles, often oversimplified into classically activated pro-inflammatory (M1) and alternatively activated anti-inflammatory (M2) types. Each state exhibits distinct effector functions and antimicrobial capabilities, whereby M1 macrophages have augmented antibacterial functionality owing primarily to their enhanced production of reactive oxygen species and antimicrobial peptides (10, 11). On the other hand, anti-inflammatory M2 macrophages act as mediators of homeostasis and tissue repair, generating anti-inflammatory cytokines, inducing collagen production, and clearing cellular debris (12–14). Numerous examples of pathogenic bacteria, including SA, have been reported to hijack macrophage polarization mechanisms in order to establish intracellular persistence (15–18).

The relationship between cellular metabolism and immune function has emerged as a critical determining factor in macrophage polarization and their antimicrobial response (19–22). This immunometabolic axis represents a complex regulatory network where metabolic pathways not only support energy requirements but also actively shape immune cell function and phenotype. Specifically, M1 macrophages reshape their metabolism to rapidly generate ATP through glycolysis to enable acute pro-inflammatory responses to infection, while M2 macrophages are reliant on the tricarboxylic acid (TCA) cycle and oxidative phosphorylation for more efficient but slower ATP generation.

Fluorescence lifetime imaging microscopy (FLIM) has emerged as a powerful tool for investigating these metabolic states in living cells, offering unique insights into dynamic host metabolic responses through the measurement of free vs protein-bound NADH (23). Compared to conventional *in vitro* endpoint assays for determining aggregate metabolic pathway utilization of a population of cells (e.g., Seahorse assays), FLIM provides metabolic information at a single-cell resolution in a non-destructive manner, affording researchers increased resolution of heterogenous metabolic responses in cellular populations without sacrificing model complexity. This is especially true in the context of macrophage polarization, which has recently gained appreciation as a highly diverse process where macrophage activation states can exist on a broad spectrum (24).

Therapeutic modulation of macrophage polarization for the treatment of various diseases has garnered intense research interest in recent years, particularly targeting cancer (25–29). It has been previously shown that various antibiotics exhibit immunomodulatory effects beyond their direct antimicrobial activity (30–36). Depending on the agent, these immunometabolic effects can prove beneficial (e.g., bedaquiline with enhanced phagolysosomal fusion capacity) or detrimental (e.g., ciprofloxacin with impaired phagocytic capacity) (35, 36).

Here, we sought to characterize the effects of antistaphylococcal antibiotics on Kupffer cell immunometabolism relative to intracellular bacterial clearance using a novel approach with FLIM combined with gene expression analysis on markers of distinct M1 and M2 metabolic pathways. Our study tests the hypothesis that antibiotics differentially modulate macrophage metabolism, which may boost or dampen macrophage clearance of intracellular *S. aureus*. We offer new insights into how these antibiotics influence both the metabolic profiles and immunological function of KCs in the context of SAB, potentially informing future therapeutic strategies to combat SAB.

## RESULTS

### Kupffer cell immunometabolism via classical stimulation and SA infection

**Fig 1:**
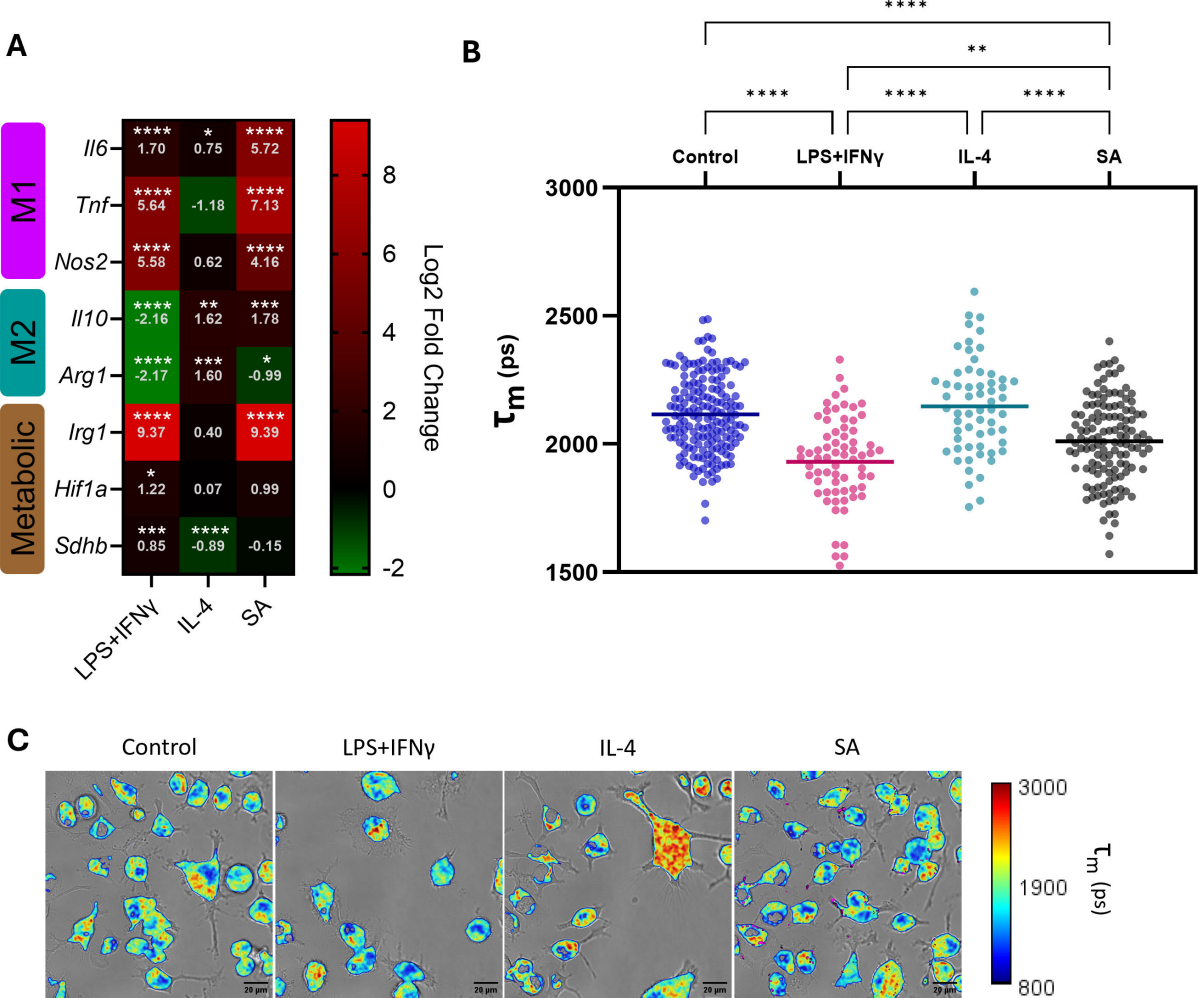
Kupffer cell (KC) metabolism without stimulation (control) and upon stimulation with LPS + IFN-γ, IL-4, or SA infection. KCs were treated with LPS (100 ng/mL) + IFN-γ (20 ng/mL) or IL-4 (20 ng/mL) for 24 h, or infected with SA for 10 h. KC polarization and metabolism were then assessed by (**A**) RT-qPCR and (**B**) TPE-FLIM to determine average single-cell NAD(P)H lifetimes. (**C**) Representative TPE-FLIM images with mean intensity-weighted lifetime signal were overlayed with transmitted light images for each condition, with SA displayed in magenta pseudocolor. Representative genes were selected to characterize M1 (*Il6*, *Tnf*, and *Nos2*) and M2 (*Il10* and *Arg1*) polarization phenotypes, as well as *Irg1*, *Hif1α*, and *Sdhb*, to investigate metabolic changes. Gene expression fold changes were determined relative to uninfected untreated KCs and log2 transformed to report log2 fold changes (control *n* = 6, treatment *n* = 3). NAD(P)H lifetimes were determined by TPE-FLIM and subsequent biexponential tail fitting of fluorescence decay. Mean single-cell intensity-weighted lifetimes (τ_*m*_) were calculated for each KC population (control *n* = 172, LPS + IFN-γ *n* = 69, IL-4 *n* = 60, SA *n* = 129). * indicates *P <* 0.05; ** indicates *P <* 0.01; *** indicates *P <* 0.001; and **** indicates *P <* 0.0001 in one-way analysis of variance with Tukey’s *post hoc* analysis.

To establish a baseline for later evaluation of antimicrobial effects on KC immunometabolism, we activated KCs through classical stimulation or infection with SA to measure the effects of polarization on immunometabolism by quantifying expression of representative genes via reverse transcription quantitative PCR (RT-qPCR) and performing two-photon excitation fluorescence lifetime imaging microscopy (TPE-FLIM) (Fig. 1A through C). M1 and M2 polarization was induced by classical treatments with lipopolysaccharide (LPS) + interferon-γ (IFN-γ) or interleukin (IL)-4, respectively, for 24 h. Separately, KCs were infected with SA for 10 h as this timepoint was previously shown in our *in vitro* intracellular infection model to differentiate the antibacterial killing activity among the tested antibiotics (37–39).

**Fig 2:**
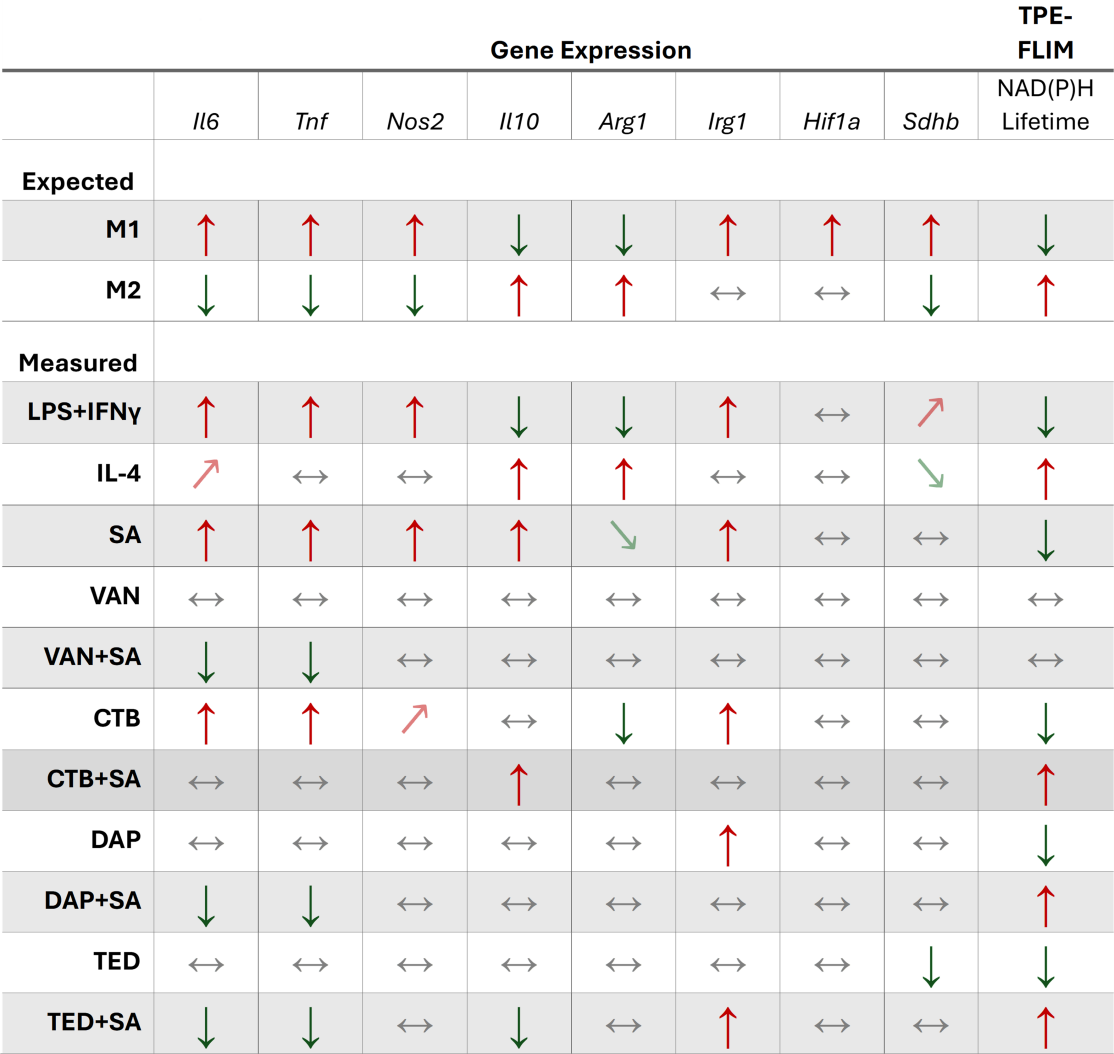
Expected gene expression and NAD(P)H lifetime responses to M1 or M2 polarization and summary of findings for antibiotic treatment and infection. Up/down arrows denote over 1-log increase or decrease in L2FC for gene expression and *P* < 0.05; arrows for lifetime denote significant (*P* < 0.05) increase or decrease in mean NAD(P)H lifetime. Slanted up/down arrows indicate under 1-log increase or decrease in L2FC for gene expression and *P* < 0.05. Horizontal bidirectional arrows indicate no change. Infected treated KC group summary trends displayed relative to infected untreated KC group (SA). CTB, ceftobiprole; DAP, daptomycin; TED, tedizolid; VAN, vancomycin.

The following genes were selected to represent M1 (*Il6*, *Tnf*, and *Nos2*) and M2 (*Il10* and *Arg1*) polarization states, as well as macrophage metabolic response (*Irg1*, *Hif1α*, and *Sdhb*) to quantify expression by RT-qPCR. The combined LPS + IFN-γ has been shown to activate multiple pro-inflammatory pathways in macrophages to induce potent pro-inflammatory cytokine expression, including IL-6 and tumor necrosis factor α as well as nitric oxide synthase 2 (NOS2) production to prioritize arginine consumption to generate antimicrobial reactive nitrogen species (40–44). On the other hand, M2 polarization is induced by the anti-inflammatory cytokine, IL-10, acting on JAK/STAT pathways to curb hyperinflammation in macrophages, while arginase one production is increased to prioritize arginine as a substrate for downstream production of proline and polyamines to be utilized in tissue repair (45, 46). Induction of *Irg1* expression results in the production of aconitate decarboxylase, which catalyzes the production of the antimicrobial metabolite itaconate and has been associated with pro-inflammatory responses (22). This metabolite has garnered major interest in the field of immunometabolism in part due to its myriad roles in different infectious disease contexts and is implicated in augmenting host responses to SA infection (47–50). *Hif1a* is responsible for the production of hypoxia-inducible factor 1α, which acts as a metabolic regulator to hypoxic response in part by promoting increased glycolysis in macrophages, contributing to M1 polarization (51, 52). The metabolic gene *Sdhb* encodes subunit B of the enzyme succinate dehydrogenase (SDH) and is an integral component of the TCA cycle and oxidative phosphorylation. While M1 macrophages are known to have a break at SDH in the TCA cycle, recent studies have highlighted the importance of SDH in generating inflammatory responses in macrophages through oxidation of succinate and reverse electron transfer (53, 54). Figure 2 summarizes our expected gene expression responses to M1 and M2 polarization, along with our observed responses to classical polarization control treatments and infection. Upon stimulation with LPS 100 ng/mL + IFN-γ 20 ng/mL for 24 h, the following changes in KC gene expression were observed compared to unstimulated control, reported as log2 fold change (L2FC) to depict upregulation and downregulation relative to baseline at zero. As expected, a significantly increased expression of genes biased toward M1 phenotype was observed: *Tnf* (L2FC = 5.64, *P* < 0.0001), *Nos2* (L2FC = 5.58, *P* < 0.0001), and *Il6* (L2FC = 1.70, *P* < 0.0001) (Fig. 1A). A reciprocal decrease among M2-associated genes was noted: *Arg1* (L2FC = −2.17, *P* < 0.0001) and *Il10* (L2FC = −2.16, *P* < 0.0001). Among the metabolic genes that are biased toward M1 polarization, increased expression was observed in descending order of magnitude: *Irg1* (L2FC = 9.37, *P* < 0.0001), followed by *Hif1a* (L2FC = 1.22, *P* = 0.0370) and *Sdhb* (L2FC = 0.85, *P* = 0.0001).

With respect to classical M2 stimulation, an expected increased expression in *Il10* (L2FC = 1.62, *P* = 0.0019) and *Arg1* (L2FC = 1.60, *P* = 0.0007) was observed. Interestingly, a slight but significant increase in *Il6* expression was noted (L2FC = 0.75, *P* = 0.0182), which other groups have recently reported as a macrophage response to IL-4 stimulation and is suggested to play a role in regulating inflammatory responses (55, 56). Of note, no significant increases in any other M1-associated genes were observed in response to IL-4 stimulation. Among the metabolic genes, IL-4 treatment led to a significant decrease in expression of *Sdhb* (L2FC = −0.89, *P* < 0.0001) and no significant changes in *Irg* or *Hif1a* (*P* = 0.6581, *P* = 0.9982, respectively). Overall, these findings are in line with expectations for these commonly used M1 and M2 stimulation controls (57–59).

In the presence of SA infection for 10 h, KCs exhibited an expected pro-inflammatory M1 phenotype, with significant increases in expression of *Il6*, *Tnf*, and *Nos2* (L2FC = 5.72, 7.13, and 4.16, respectively; *P* < 0.0001). SA infection also significantly decreased *Arg1* expression (L2FC = −0.99, *P* = 0.0145) but increased *Il10* expression (L2FC = 1.78, *P* = 0.0009). The observed elevated *Il10* expression, despite a predominant pro-inflammatory profile, likely represents a compensatory response to the extensive pro-inflammatory signaling due to the acute infection, a pattern consistent with previous reports showing macrophages can increase IL-10 production as a regulatory mechanism to mitigate inflammation-induced tissue damage (60, 61). Similar to the LPS + IFN-γ treatment group, infected KCs also had a substantial increase in *Irg1* expression (L2FC = 9.39, *P* < 0.0001).

FLIM was performed to assess the effects of macrophage M1/M2 classical activation as well as infection on KC. Shorter average NAD(P)H lifetime is indicative of an increase in utilization of glycolysis for cellular energy—a metabolic feature of M1 macrophages—and LPS + IFN-γ treatment induced a significant decrease in average single-cell NAD(P)H fluorescence lifetime in picoseconds compared to the control group (1,930 ps vs 2,115 ps; *P* < 0.0001), with a similar trend observed among KCs 10 h post-SA infection (2,011 ps; *P* < 0.0001) (Fig. 1B and C). On the other hand, IL-4 treatment did not induce significant changes in single-cell NAD(P)H lifetime.

### Antibiotic exposure modulates Kupffer cell metabolism in a context-dependent manner

**Fig 3:**
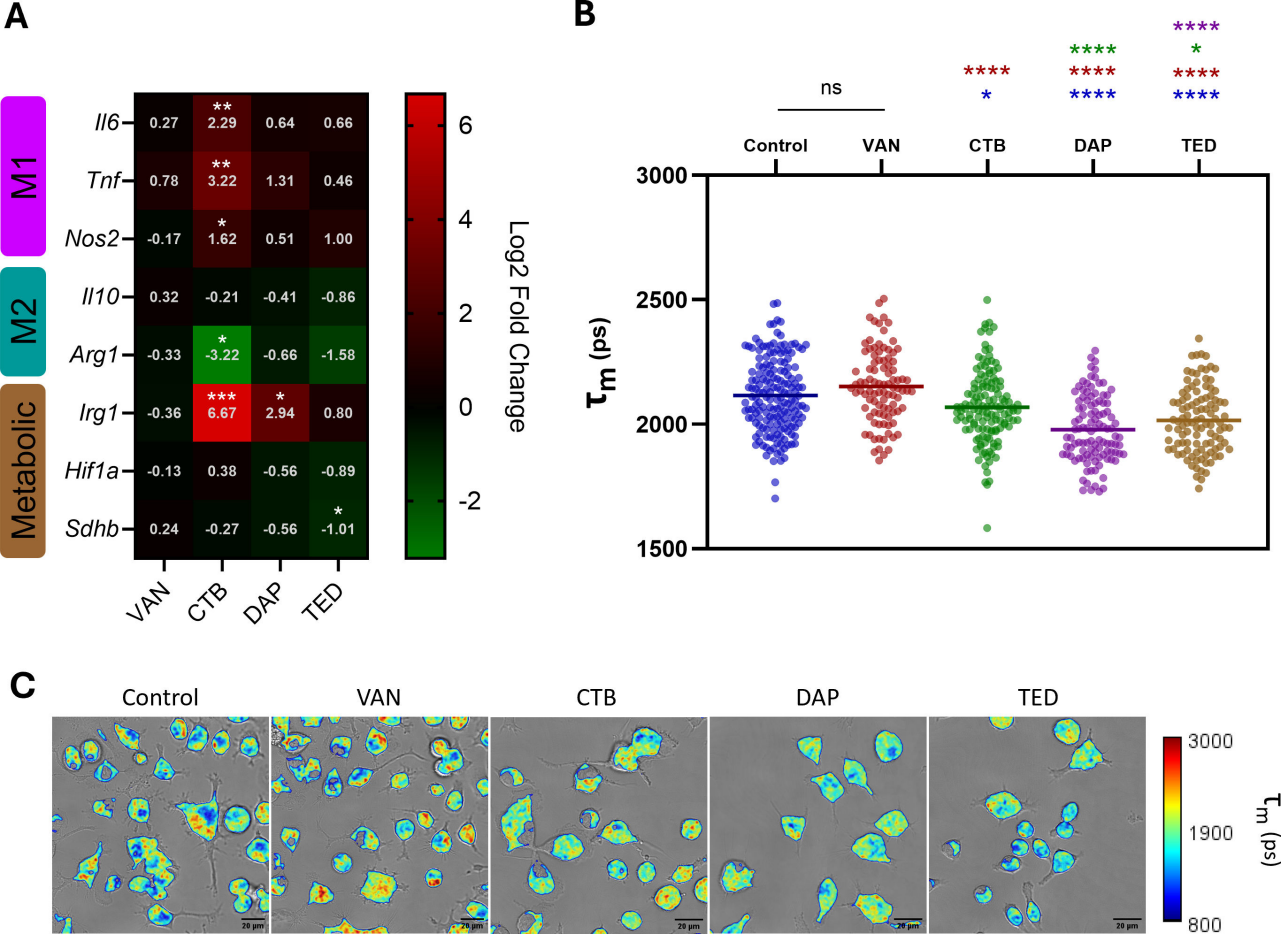
Kupffer cell metabolism upon antibiotic exposure in the absence of infection. KCs were treated with antibiotics at reported human *C*_max_ concentrations for 10 h. M1 (*Il6*, *Tnf*, and *Nos2*) and M2 (*Il10* and *Arg1*) polarization, as well as metabolic genes (*Irg1*, *Hif1α*, and *Sdhb*), were assessed by RT-qPCR (**A**), and NAD(P)H fluorescence lifetime was determined by TPE-FLIM (**B**). Fluorescence decay curves from generated NAD(P)H data acquired by TPE-FLIM from each region of interest were fitted to a biexponential decay function and exported to ImageJ for single-cell NAD(P)H lifetime analysis. Spatial NAD(P)H lifetime data were further processed, then overlaid with associated transmitted light images to generate representative FLIM images for each treatment group (**C**). Gene expression fold changes determined relative to untreated KCs and log2 transformed to report log2 fold changes (*n* = 3). Images containing NAD(P)H fluorescence lifetime data were extracted to calculate mean single cell intensity-weighted lifetimes (τ_*m*_) for each treatment population (control *n* = 172, VAN *n* = 95, CTB *n* = 135, DAP *n* = 107, TED *n* = 105). * indicates *P* < 0.05, ** indicates *P <* 0.01, *** indicates *P <* 0.001, and **** indicates *P <* 0.0001 in one-way analysis of variance (ANOVA) with Dunnet’s *post hoc* analysis for gene expression data and one-way ANOVA with Tukey’s *post hoc* analysis for NAD(P)H lifetime data.

We first assessed whether antistaphylococcal agents representing different pharmacologic classes alter KC immunometabolism at baseline (without infection) by performing RT-qPCR and FLIM following 10 h exposure to vancomycin (VAN), ceftobiprole (CTB), daptomycin (DAP), and tedizolid (TED) at reported human *C*_max_ concentrations (Fig. 3A through C). We then compared the immunometabolic effects on SA-infected KCs among the agents tested to examine how these effects are linked to clearance of intracellular SA.

Figure 2 shows the context-dependent effects of antibiotics on KC gene expression and NAD(P)H lifetime with and without SA infection. In the absence of infection, VAN imparted no change in gene expression or NAD(P)H lifetime, while DAP and TED treatments affected only expression of selected KC genes but significantly reduced mean NAD(P)H lifetime toward a glycolytic trajectory. Notably, exposure of KCs to CTB without infection induced a clear shift toward M1-associated gene expression profile exhibited by significant upregulation in all measured representative M1 genes (*Tnf*, *Il6*, *Nos2*, and *Irg1*), with a corresponding decrease in *Arg1* expression along with a significant decrease in NAD(P)H lifetime (Fig. 3A). Taken together, both measures of polarization gene expression and NAD(P)H metabolic utilization indicated that CTB, DAP, and TED affected KCs toward an M1 phenotype and/or glycolytic metabolic trajectory, while VAN exerted no effects on KC immunometabolism in the absence of infection.

**Fig 4:**
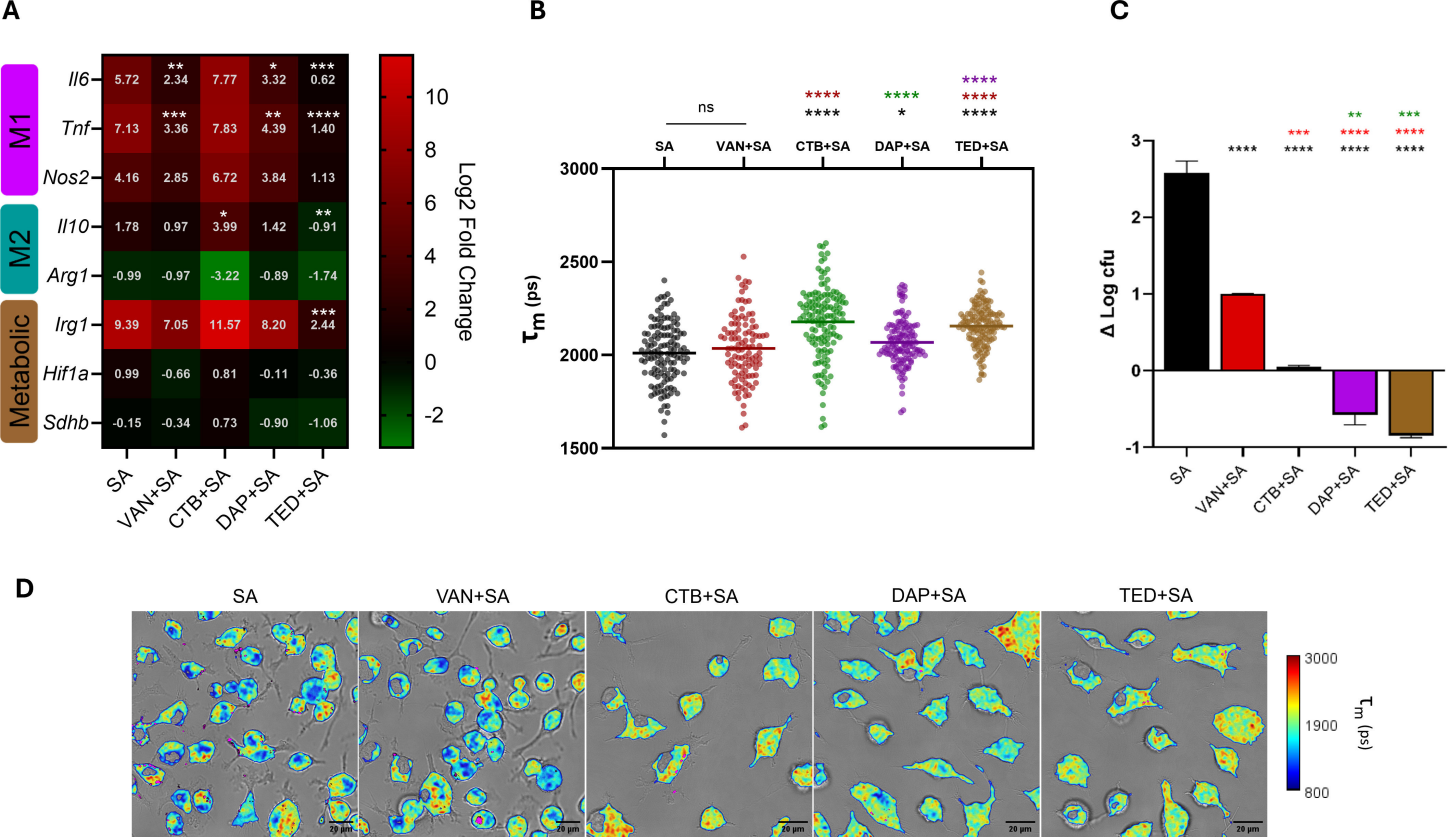
Effect of infection and antibiotic treatment on KC metabolism. KCs were treated with antibiotics at reported human *C*_max_ concentrations for 10 h in the presence of established infection with gfp USA300 LAC. Infection was established with a multiplicity of infection of 5 of gfp SA before brief treatment with gentamicin to eradicate any remaining extracellular SA before introducing antibiotic-supplemented media at reported human *C*_max_ concentrations for 10 h. (**A**) KC polarization and metabolic alterations were assessed by RT-qPCR and (**B**) TPE-FLIM. (**C**) As before, following biexponential fitting of NAD(P)H fluorescence decay curves, images containing mean intensity-weighted lifetime data were extracted, and average single-cell NAD(P)H lifetimes for each KC population were determined. Viable intracellular CFUs were also determined under identical infection and treatment conditions to compare the intracellular antibacterial efficacy of each antibiotic treatment. (**D**) Representative FLIM images with spatial NAD(P)H lifetime data overlaid with associated transmitted light images and gfp signal (pseudocolor magenta) were also generated for each treatment group for infected KCs. qPCR experiments were performed in triplicate. TPE-FLIM experiments were performed at least in duplicate (SA *n* = 129, VAN + SA *n* = 108, CTB + SA *n* = 134, DAP + SA *n* = 130, TED + SA *n* = 143). Intracellular killing experiments were performed at least in duplicate. * indicates *P* < 0.05; ** indicates *P <* 0.01; *** indicates *P <* 0.001; and **** indicates *P <* 0.0001, color coded where applicable to denote significant differences between associated treatment groups, in one-way ANOVA with Dunnet’s *post hoc* analysis for gene expression data and one-way ANOVA with Tukey’s *post hoc* analysis for NAD(P)H lifetime and Δ log CFU data.

To investigate the effects of antistaphylococcal antibiotics on KC polarization in the context of intracellular infection, we profiled KC gene expression following infection with SA and 10 h post-antibiotic treatment compared to the infected untreated group (Fig. 4A through C). Across the different antibiotic treatment groups, distinct immunometabolic effects on KCs with respect to gene expression were observed (Fig. 2 and 4A). Upon infection, VAN and DAP dampened expression of M1-associated genes (*Tnf* and *Il6)*. While VAN and DAP treatment did not alter M2-associated expression of *Il10* or *Arg1*, TED treatment showed the most pronounced anti-inflammatory effect on infected KCs, significantly suppressing infection-induced pro-inflammatory *Tnf* and *Il6* expression (L2FC = 1.40 vs 7.13, *P <* 0.0001, and L2FC = 0.625 vs 5.72, *P* = 0.0002, respectively), as well as *Irg1* (L2FC = 2.44 vs 9.39, *P =* 0.0007) and *Il10* (L2FC = −0.91 vs 1.78, *P* = 0.0024) expression, with the latter potentially signifying sufficient attenuation of infection-driven inflammation by TED treatment. Interestingly, CTB treatment retained the pro-inflammatory profile of infected KCs but significantly increased *Il10* expression (L2FC = 3.99 vs 1.78, *P =* 0.016) (Fig. 4A). Changes in expression of the metabolic *Hif1a* and *Sdhb* genes for the untreated infected KC control remained the same despite antibiotic treatment.

We then examined the metabolic impact of VAN, CTB, DAP, and TED on infected KCs by FLIM. Compared to the infected untreated group, VAN treatment of infected KCs did not alter the metabolic state as shown by similar NAD(P)H lifetime between conditions (2,036 ps vs 2,011 ps; *P =* 0.7326), while treatment with DAP, TED, and CTB exhibited an overall increase in mean NAD(P)H lifetimes to varying degrees (Fig. 2 and 4B and D). DAP treatment of infected KCs, which showed a dampened M1 expression profile, exhibited a modest increase in NAD(P)H lifetime when compared to untreated infected KCs (2,068 ps; *P* = 0.0244). Gene expression and NAD(P)H lifetime data for TED-treated infected KCs were in agreement, where the most dampened pro-inflammatory expression profile and a pronounced increase in mean NAD(P)H lifetime (2,155 ps; *P* < 0.0001) were observed. Interestingly, while CTB treatment of infected KCs led to a sustained M1 expression profile, the mean single-cell NAD(P)H lifetime was prolonged compared to untreated infected KCs (2,178 ps vs 2,011 ps; *P <* 0.0001), suggesting other factors in the context of infection may be contributing to the observed increase in bound NAD(P)H. Furthermore, a considerably heterogenic response in bound NAD(P)H (SD = 192, range = 986 ps) was observed in the CTB-treated infected KC population compared to other antibiotic treatment groups (Fig. 4B). Taken together, M1/M2 gene expression data and NAD(P)H lifetime measures suggest variable antibiotic-mediated responses upon infected KCs among CTB, DAP, and TED, while VAN blunted pro-inflammatory gene expression but did not alter the glycolytic state of KCs.

To determine the correlation between gene expression, NAD(P)H lifetime data, and intracellular killing efficacy of these antibiotics, we enumerated intracellular SA within infected KCs following the same 10 h antibiotic treatment conditions to determine the difference in log CFU relative to initiation of infection (Fig. 4C). Intracellular bacterial eradication in KCs is expected to reduce the level of PAMPs, thereby leading to reduced pro-inflammatory response and longer NAD(P)H lifetime toward resolving M2 activated state; however, this was not observed with VAN and CTB treatments. Despite VAN treatment after 10 h, a 1-log intracellular CFU increase relative to the start of infection was observed, corresponding to a significantly blunted pro-inflammatory gene expression profile, along with a relatively unchanged NAD(P)H lifetime profile when compared to the infected untreated KC group. In contrast, with CTB treatment, an increase in intracellular SA population-wide was effectively halted, with only a 0.05 log CFU increase (*P* < 0.0001), while pro-inflammatory expression levels were retained, yet NAD(P)H lifetime increased significantly. It is notable that the contribution of individual cells to this global intracellular bacterial count was likely uncovered by FLIM as exhibited by the extreme heterogeneity in single-cell NAD(P)H lifetime in the CTB treatment group. DAP and TED treatment both led to significant reductions in intracellular bacterial load after 10 h treatment (Δ log CFU = −0.58, *P* < 0.0001; Δ log CFU = −0.85, *P* < 0.0001, respectively) and corresponding increases in mean NAD(P)H. Collectively, the above findings indicate variable trajectories in gene expression and NAD(P)H lifetime data in relation to intracellular antimicrobial efficacy following treatment with antibiotics from different pharmacologic classes, with an overall increase in mean NAD(P)H lifetime corresponding to bacterial eradication.

**Fig 5:**
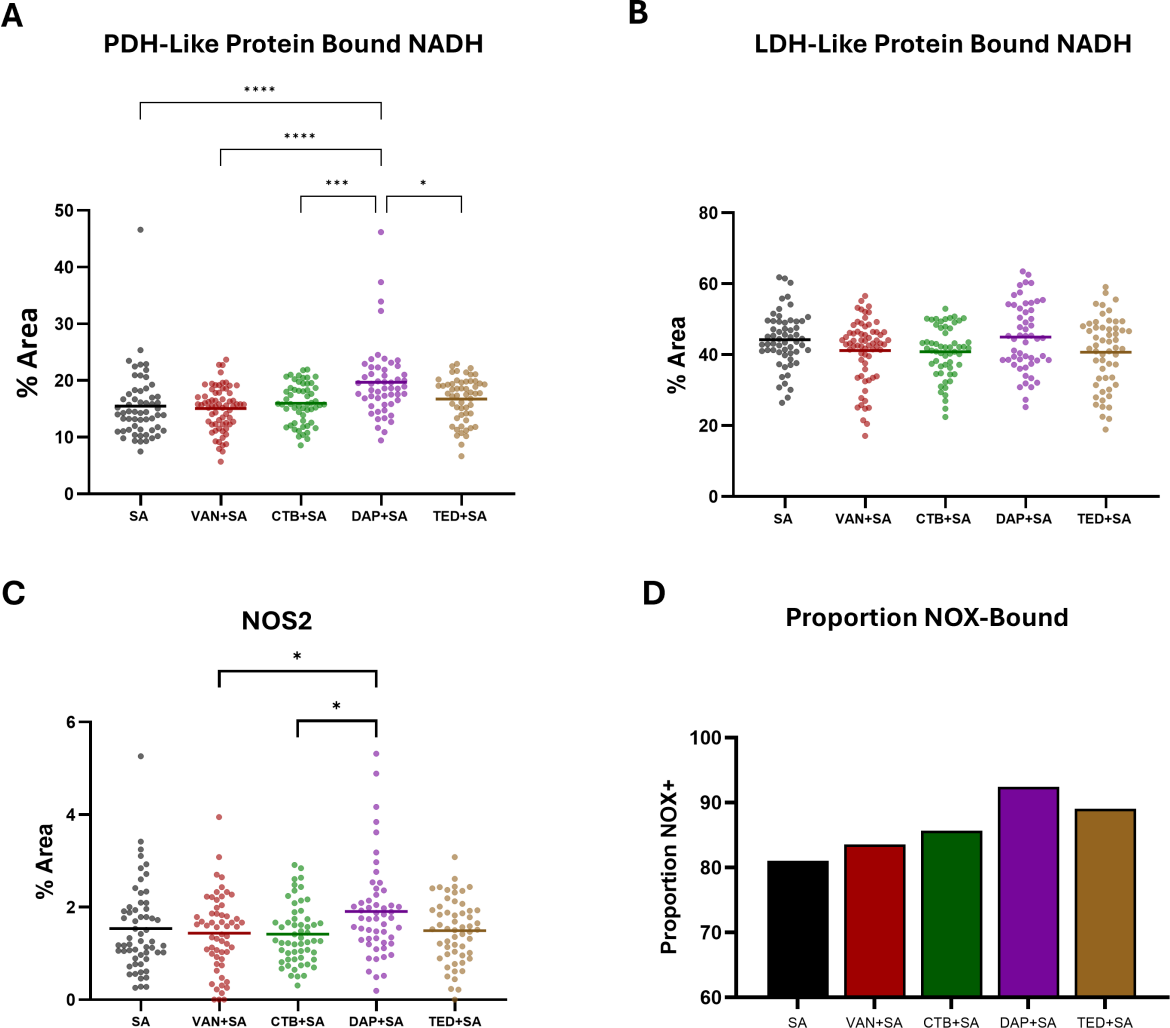
Metabolic mapping of NAD(P)H lifetimes among infected KC population with and without antibiotic treatment. NAD(P)H lifetimes were separated and characterized to investigate treatment-induced changes to NADH bound to pyruvate dehydrogenase (PDH)-like enzymes (2.17–2.60 ns, generally associated with oxidative phosphorylation) (**A**), lactate dehydrogenase (LDH)-like enzymes (1.24–2.005 ns, generally associated with anaerobic glycolysis) (**B**), NOS2 (2.55 ns) (**C**), as well as activated NADPH oxidase (NOX2) (3.65 ns) (**D**). Percent area mean fluorescence lifetime for PDH-like, LDH-like, and NOS2 enzymes was determined. (SA *n* = 58, VAN + SA *n* = 67, CTB + SA *n* = 56, DAP + SA *n* = 53, TED + SA *n* = 55). * indicates *P* < 0.05; *** indicates *P <* 0.001; and **** indicates *P <* 0.0001 in one-way ANOVA with Tukey’s *post hoc* analysis.

We further stratified the single-cell mean lifetime data to provide a metabolic map of NAD(P)H utilization among antibiotic-treated infected KCs by grouping NAD(P)H-dependent enzymes generally associated with glycolysis (lactate dehydrogenase [LDH]-like enzymes 1.24–2.005 ns) or oxidative phosphorylation (pyruvate dehydrogenase [PDH]-like enzymes 2.17–2.60 ns) (62). We also determined NAD(P)H utilization among two enzymes associated with immune function: activated NADPH oxidase (NOX2) (3.65 ns), which contributes to phagolysosomal respiratory burst, and NOS2 (2.55 ns) (63). Both of these enzymes are upregulated in M1 macrophages (44, 64). We then compared the percent area mean fluorescence lifetime for each of these parameters. We observed a significant increase in PDH-like protein-bound NADH among DAP-treated infected KCs only (19.7% vs 15.5%, *P* < 0.0001) compared to untreated infected KC control and compared to all other treatments (VAN: *P* < 0.0001, CTB: *P* = 0.0007, and TED: *P* = 0.0133) (Fig. 5A). However, no significant difference in mean single-cell percent area was observed for LDH-like protein-bound NADH (Fig. 5B). For NOS2 percent area, we observed significant differences between DAP-treated infected KCs compared to CTB and VAN treatment groups (1.91 vs 1.42%, *P* = 0.0206; 1.91 vs 1.44%, *P* = 0.025, respectively) (Fig. 5C). When evaluating the proportion of cells with NADPH NOX2 binding based on fluorescence lifetime, we also observed the greatest increase in DAP-treated infected KCs (92.5%) compared to all other groups (SA: 81.0%, VAN: 83.6%, CTB: 85.7%, and TED: 89.1%) (Fig. 5D). Overall, stratification and subsequent analysis of NAD(P)H lifetimes among antibiotic treatment groups suggest potential involvement of discrete NAD(P)H-mediated metabolic effectors that may contribute to antibiotic-mediated clearance of intracellular SA, with the largest proportion of NOX2 binding observed corresponding to the greatest intracellular killing among DAP and TED treatment groups.

## DISCUSSION

Despite antibiotic therapy, *S. aureus* bacteremia remains a significant clinical challenge with persistent bloodstream infection contributing to high mortality rates. While KCs provide critical host defense early in SAB, they also serve as potential reservoirs of SA, which supports persistence. Thus, a deeper understanding of potential indirect effects of antibiotics on KC immune function is necessary to better target this pathogenic reservoir. Others have described antibiotic effects on immune cell metabolism and function, both positive (e.g., bedaquiline improves phagolysosomal antimicrobial function) and negative (ciprofloxacin reduces phagocytic capacity) (35, 36). In the context of SAB, antibiotic-specific effects on KC metabolism and function remain largely unexplored. To address this, we investigated the impact of antistaphylococcal antibiotics (VAN, CTB, DAP, and TED) on KC immunometabolism during SA infection using novel approaches combining FLIM metabolic imaging with gene expression analysis of KC polarization.

Our findings revealed distinct immunometabolic signatures of antistaphylococcal antibiotics on KC function, characterized by both transcriptional and NAD(P)H utilization changes that vary significantly between the tested antibiotics. CTB demonstrated strongest alignment between induction of a pro-inflammatory M1-like gene expression profile in uninfected KCs and a simultaneous decrease in NAD(P)H lifetime, suggesting a shift in metabolic trajectory indicative of classical M1 macrophage polarization. The effects of CTB on macrophage immunometabolism suggest a potential mechanism beyond its direct antimicrobial activity, which likely contributes to its intracellular bacterial clearance amounting to 1.06 log CFU decrease from baseline following 24 h treatment, as previously observed (37). Beneficial effects of beta-lactam agents on host immune response have been previously reported. Volk et al. described an association between higher pro-inflammatory cytokines among SAB patients treated with beta-lactams and increased survival compared to vancomycin and daptomycin (65). Others reported beta-lactams enhancing innate immune-mediated activity against SA by increasing host defense peptide binding to the bacterial membrane (66).

It is notable that the observed metabolic shifts of KCs in the context of infection and antibiotic treatment as indicated by NAD(P)H lifetime changes do not necessarily align with gene expression findings, which highlights the heterogenous response to infection and antibiotic treatment when measured at the single-cell level using the TPE-FLIM approach. CTB treatment induced prominent heterogeneity among infected KCs, with a longer mean NAD(P)H lifetime compared to uninfected control KCs as well as uninfected CTB-treated KCs. In contrast, DAP and TED induced the greatest shifts in NAD(P)H metabolic signatures toward glycolytic trajectory yet exhibited only a weak positive trend in pro-inflammatory M1 expression in the absence of infection. These findings suggest a more complex association between KC polarization and NAD(P)H metabolic utilization. Furthermore, in the context of infection, our findings suggest a distinct modulation of KC metabolism by DAP and TED, characterized by an observed increase in mean NAD(P)H lifetime that correlates with their suppression of pro-inflammatory gene expression. A potential explanation to this observation may lie in the efficiency by which DAP and TED exerted rapid killing of intracellular SA resulting in lower bacterial burden and thus lower PAMP-mediated signaling within the host cells. In addition, TED exhibits antivirulence properties against SA and may dampen virulence-mediated host inflammatory responses to intracellular SA (67). Of particular interest is the observation of a VAN-driven blunting of pro-inflammatory KC phenotype paired with an unchanged NAD(P)H fluorescence lifetime relative to its inability to control intracellular infection in our model. In contrast, all other antibiotic treatments led to a significant increase in mean NAD(P)H lifetime and were effective in controlling the intracellular infection. Furthermore, we and others have characterized the relative intracellular accumulation of VAN, CTB, DAP, and TED in macrophages, with VAN exhibiting greater intracellular accumulation relative to CTB and DAP (37–39). Thus, relative differences among antibiotic accumulation are not sufficient to explain observed differences in intracellular bacterial killing efficacy or immunometabolic effects. The tested antibiotics differ in gene expression trends yet share a similarity in the association of increased NAD(P)H lifetimes with intracellular antibacterial efficacy. With markedly reduced PAMPs at this stage of infection due to CTB, DAP, and TED’s superior intracellular antibacterial efficacy, the metabolic shift toward a more OXPHOS trajectory may indicate the beginning of infection resolution responses in KCs as they attempt to mitigate potential harmful consequences of a prolonged inflammatory state. As described by Watson et al., tightly calibrated pro-inflammatory and resolution responses in host immunity are crucial to successful outcomes in many infectious diseases (68). They posited that therapeutics that enhance these “pauci-inflammatory” host microbicidal responses may be beneficial in maximizing intracellular bacterial clearance while also minimizing potential collateral damage from hyperinflammation. Collectively, we have shown here differential effects of antistaphylococcal antibiotics on KC immunometabolism, with observed increases in NAD(P)H lifetime for CTB, DAP, and TED, perhaps signifying early stages of infection resolution, whereas NAD(P)H lifetime was not altered and intracellular infection continues unchecked under VAN treatment.

FLIM techniques for measuring NAD(P)H are sensitive in distinguishing free vs protein-bound NAD(P)H in the intracellular environment to characterize overall metabolic states; however, others have recently begun expanding its use to gain more granular insights into which proteins NAD(P)H binds (62, 69, 70). This enhanced resolution thus provides opportunities for further investigation of electron carrier utilization within the wider immunometabolic framework in the context of infection. Our stratification of NAD(P)H protein binding suggested relatively high anaerobic glycolysis metabolic utilization in the context of SA infection exhibited by elevated LDH-like enzymatic contribution to overall NAD(P)H lifetime signal. Similar uniformity among PDH-like signal contribution across treatments was observed, except with DAP treatment, which was associated with significant increases in PDH-like enzymatic activity. Furthermore, DAP treatment elicited a significant increase in inducible nitric oxide synthase-associated fluorescence lifetime compared to other antibiotics in the presence of infection. High proportions of KCs with activated NOX were also observed across all treatments as the KCs attempted to eradicate the intracellular SA, with higher proportion of NOX positivity trending with efficacy of intracellular eradication. This metabolic stratification of NAD(P)H FLIM signals highlights the differential effects of DAP on the metabolic response of infected KCs but does not fully explain metabolic differences seen among the tested antibiotics. Further efforts to elucidate antibiotic modulation of these potential NADH and NADPH binding targets will need to be done in this regard.

Understanding which antibiotics may enhance or dampen innate immune responses to infection could offer valuable insights to clinicians when making treatment decisions. Others have also demonstrated the ability of antibiotics to modulate macrophage polarization. Haydar et al. found that azithromycin induces M2 polarization in macrophages by inhibiting inflammatory STAT1 and NF-κB pathways, while He and Marneros showed that doxycycline can inhibit M2 polarization in macrophages (71, 72). Depending on the disease context, these effects on macrophage polarization could potentially contribute to patient outcomes in opposite directions. Thus, it is important to further our understanding of the potential effects of antibiotics on host immune cells.

Our study has several limitations. First, the use of KCs in our *in vitro* model does not fully represent the *in vivo* infection condition as the cells were removed from the sinusoidal microphysiological environment from which they reside. KCs line the liver sinusoid and interact with liver sinusoidal endothelial cells as well as hepatic stellate cells to orchestrate innate immune responses to bacterial bloodstream infection (73–75). How these cells interact and communicate within this microenvironment may alter their immunometabolic state and drive infection outcomes during SAB. Nonetheless, our current findings highlight correlations between KC polarization and NAD(P)H utilization to provide new insights into the mechanistic links between antibiotic-induced metabolic changes and immune function in KCs. The distinct patterns observed with different antibiotics suggest that these compounds may preferentially influence specific aspects of cellular metabolism, subsequently shaping the immune response and infection outcome.

Collectively, our findings have potential implications for therapeutic strategies in SAB, particularly in cases of persistent infection where standard approaches often fail. The distinct immunomodulatory profiles of antibiotics from different pharmacologic classes allow for the precision selection of agents to treat SAB based on host-directed immune activating effects along with bacterial susceptibility.

## MATERIALS AND METHODS

### Bacterial growth conditions

USA300 LAC glycerol stocks were streaked onto tryptic soy agar (TSA) plates and incubated at 37°C overnight, then three representative CFUs were subcultured in 5 mL tryptic soy broth the next day. The culture was then placed in an incubator with shaking at 37°C, 250 rpm, and incubated for 16–20 h prior to use in *in vitro* assays.

### Cell culture conditions

An immortalized KC line established from C57BL/6 mice was used in this study (76). KCs were maintained in Dulbecco's modified Eagle medium (DMEM) (Thermo Fisher, Waltham, MA, USA) + 10% FBS (Genesee Scientific, Morrisville, NC, USA) + 10 µg/mL bovine insulin (Sigma-Aldrich, Missouri, USA) + 250 µM monothioglycerol (Sigma-Aldrich). For gene expression and FLIM assays, the same medium was utilized, with supplementation of the tested antibiotics where applicable.

### Antibiotic preparation

Antistaphylococcal antibiotics, which are currently prescribed or recently approved for the treatment of SAB, were chosen for evaluation in this study. Vancomycin (Sigma-Aldrich), daptomycin (MedChemExpress, New Jersey, USA), ceftobiprole (TargetMol, Massachusetts, USA), and tedizolid (MedChemExpress) were purchased from commercially available sources and prepared and stored according to manufacturer recommendations.

### Gene expression measurement of KC polarization and immunometabolism by RT-qPCR

KCs were seeded into 24-well tissue culture plates at 3 × 10^5^ cells/well in prepared DMEM as previously described and incubated overnight at 37°C with 5% CO_2_. Establishment of infection was performed as previously described (34). Briefly, KCs were washed with phosphate-buffered saline (PBS), infected with SA at a multiplicity of infection (MOI) of 5 for 1.5 h at 37°C with 5% CO_2_, then washed and incubated with gentamicin at 100 µg/mL for 2 h to eradicate the remaining extracellular SA. Then media supplemented with antibiotics at human *C*_max_ concentrations were added, and KCs were incubated for 10 h at 37°C with 5% CO_2_. KC RNA was then extracted using an RNeasy Mini column-based extraction kit (Qiagen, Limburg, Netherlands) before further concentration with Zymo Clean and Concentrate Kits (Irvine, CA, USA). RNA was then reverse transcribed to cDNA with Bio-Rad iScript cDNA synthesis kits (Hercules, CA, USA). Primers were synthesized and purchased from Integrated DNA Technologies (Cedar Rapids, IA, USA). Primer sequences are listed in Table 1.

**TABLE 1:** PCR primers used for RT-qPCR gene expression analysis

Gene	Primer sequence
*Il6*	F, GCC TTC TTG GGA CTG ATG CT R, AGC CTC CGA CTT GTG AAG TG
*Tnf*	F, CTA TGT CTC AGC CTC TTC TCA TTC R, GAG TAG ACA AGG TAC AAC CCA TC
*Nos2*	F, CTA TGG CCG CTT TGA TGT GC R, TTG GGA TGC TCC ATG GTC AC
*Il10*	F, GTT ACT TGG GTT GCC AAG R, TTG ATC ATC ATG TAT GCT TC
*Arg1*	F, CTC CAA GCC AAA GTC CTT AGA G R, AGG AGC TGT CAT TAG GGA CAT C
*Irg1*	F, GCG AAC GCT GCC ACT CA R, ATC CCA GGC TTG GAA GGT C
*Hif1α*	F, TGC TCA TCA GTT GCC ACT TC R, TGG GCC ATT TCT GTG TGT AA
*Sdhb*	F, ACC CCT TCT CTG TCT ACC G R, AAT GCT CGC TTC TCC TTG TAG

### TPE-FLIM

KCs were seeded in Ibidi µ-Slide 8-well glass bottom cover slip chamber slides at 4 × 10^4^ cells per chamber and allowed to adhere overnight. The next day, infection and respective treatments were introduced as described above. At the indicated timepoint (10 h post-infection and antibiotic treatment, or 24 h post-polarization control treatment), KCs were briefly washed in PBS and overlaid with L-15 imaging media immediately prior to TPE-FLIM imaging. NAD(P)H lifetime imaging was performed on an SP8 laser scanning confocal microscope (Leica Microsystems) equipped with a pulsed (80 MHz, 100 fs pulse width) titanium–sapphire laser and acquired with a ×63 objective lens. To measure NAD(P)H autofluorescence, TPE was utilized at 740 nm at 9.5% laser power, with subsequent detection at 450–550 nm. Time-correlated single photon counting was performed with a hybrid detector coupled to the NDD port of the SP8, with an image size of 512 × 512 pixels, 10 frame repetitions per region of interest, and a pixel dwell time of 3.16 µs. To visualize SA in the samples, TPE at 920 nm was performed in sequence immediately after NAD(P)H imaging with identical scan rate and repetition settings, and emission was detected from 490 to 555 nm.

### NAD(P)H lifetime image analysis

After image acquisition, all images were processed in LASX software by pixel binning (pixel bin = 2) and intensity thresholding to 15 counts to remove background noise. Fluorescence decay curves were then fitted to a biexponential function to determine intensity-weighted mean NAD(P)H lifetime (τ_*m*_). Biexponential fitting was determined to be adequate with χ^2^ values near 1.0 (Fig. S1). Images containing τ_*m*_ data were then exported for further analysis in Fiji (77). Cell masks were generated from the images using CellPose3 with pretrained cyto3 model and deblur image restoration, then imported into Fiji for cellular segmentation of τ_*m*_ data (78, 79). Lifetime images were further processed in Fiji to remove any 0 values from cells that were below the threshold count, as well as any pixels that exceeded the fitting range. Mean intensity-weighted lifetimes were then recorded for individual cells from each image for all treatment groups. Representative lifetime images were generated by applying a LUT and smoothing to the lifetime channel of the image, then overlaying with the associated transmitted light images, and in the case of infection groups, gfp SA signal was pseudocolored magenta and overlaid as well. For stratification of NAD(P)H lifetime signal to investigate enzymatic contribution, a subset of FLIM images from each infection group was analyzed in Fiji by applying a custom LUT set to segment lifetime values to differentiate LDH-like (1.2–2.05 ns), PDH-like (2.16–2.61 ns), NOS2 (2.52–2.58 ns), and NOX2 (3.6–3.7 ns) enzymatic binding. Percent area signal contribution per cell was then measured in Fiji for analysis. For NOX2 enzymatic binding, positivity was determined by the presence or absence of lifetime signal in each cell within the specified range.

### Intracellular killing assays

Intracellular killing assay was performed as previously described (37). Briefly, KCs were seeded into 48-well flat-bottom tissue culture plates at 1.5 × 10^5^ cells/well in prepared DMEM as previously described and incubated overnight at 37°C with 5% CO_2_. The next day, liquid gfp USA300 LAC cultures were washed and diluted prior to inoculation of KC cultures at an MOI of 5 for 1.5 h at 37°C with 5% CO_2_. Following the infection period, KCs were washed and incubated with gentamicin (Sigma-Aldrich) at 100 µg/mL for 2 h to eradicate the remaining extracellular SA. Then, KCs were washed and incubated with antibiotics at human *C*_max_ concentrations for 10 h at 37°C with 5% CO_2_. Finally, KCs were washed and lysed with 100 µL 0.1% Triton X-100 in water for 5 min at room temperature, then lysates were serially diluted in PBS and plated on TSA for enumeration of intracellular CFU.

### Statistical analysis

RT-qPCR assays were performed in triplicate using ddCt data analysis techniques in relation to uninfected untreated KC groups, and results were reported as L2FC. One-way analysis of variance (ANOVA) with Dunnett’s *post hoc* analysis was used to determine the statistical significance of differences in expression among treatment groups for each gene in relation to uninfected or infected control groups. For TPE-FLIM assays, at least three ROIs were measured for each treatment group, and one-way ANOVA with Tukey’s *post hoc* analysis was used for all TPE-FLIM results to compare differences in mean population NAD(P)H lifetime for all treatment groups. Intracellular killing assays were performed at least in duplicate, and statistical differences among treatment groups were determined using one-way ANOVA with Tukey’s *post hoc* analysis.

## SUPPLEMENTAL MATERIAL

Fig. S1 mbio.02124-25-s0001.docx Representative chi-squared evaluation for biexponential fitting NAD(P)H fluorescence decay curves.

ASM does not own the copyrights to Supplemental Material that may be linked to, or accessed through, an article. The authors have granted ASM a non-exclusive, world-wide license to publish the Supplemental Material files. Please contact the corresponding author directly for reuse.
