# Supplementary material for: Effect of antibiotics on Kupffer cell immunometabolism relative to intracellular killing of S. aureus using NAD(P)H fluorescence lifetime imaging
Source: mBio. 2025 Sep 30;16(11):e02124-25. doi: 10.1128/mbio.02124-25 (PMC12607893; doi:10.1128/mbio.02124-25)
Supplement: Fig. S1 — Representative chi-squared evaluation for biexponential fitting NAD(P)H fluorescence decay curves. [file mbio.02124-25-s0001.docx]

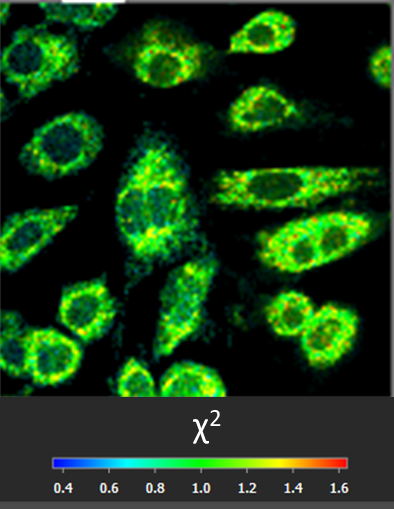


**Figure S1.** Representative image illustrating χ^2^ values following biexponential tail fitting. After performing biexponential tail fitting on fluorescence decay curves generated from the image, χ^2^ values for each pixel were visualized using a heatmap color scheme, with values near 1.0 (theoretical perfect model fit) in green.
